# Temporal dynamic of malaria in a suburban area along the Niger River

**DOI:** 10.1186/s12936-017-2068-5

**Published:** 2017-10-23

**Authors:** Mahamadou Soumana Sissoko, Kourane Sissoko, Bourama Kamate, Yacouba Samake, Siaka Goita, Abdoulaye Dabo, Mama Yena, Nadine Dessay, Renaud Piarroux, Ogobara K. Doumbo, Jean Gaudart

**Affiliations:** 1 Department of Epidemiology of Parasitic Diseases-Faculty of Medicine and Dentistry-Faculty of Pharmacy, Malaria Research and Training Centre, University of Sciences, Techniques and Technologies of Bamako, Bamako, Mali; 2 Direction Nationale de l’Hydraulique, Bamako, Mali; 3 Nadine Dessay, UMR 228 ESPACE-DEV (IRD, UM, UG, UA, UR), Responsable équipe Observation Spatiale de l’Environnement (OSE), Maison de la Télédétection, 500 rue Jean-François Breton, 34093 Montpellier Cedex 5, France; 4 Service de Parasitologie-Mycologie Hôpital de la Timone et UMR MD 3 Aix-Marseille Université, Marseille, France; 5 Aix Marseille Univ, IRD, INSERM, SESSTIM, 13005 Marseille, France

**Keywords:** Malaria, Transmission heterogeneity, Environmental risk factors

## Abstract

**Background:**

Even if rainfall and temperature are factors classically associated to malaria, little is known about other meteorological factors, their variability and combinations related to malaria, in association with river height variations. Furthermore, in suburban area, urbanization and growing population density should be assessed in relation to these environmental factors. The aim of this study was to assess the impact of combined environmental, meteorological and hydrological factors on malaria incidence through time in the context of urbanization.

**Methods:**

Population observational data were prospectively collected. Clinical malaria was defined as the presence of parasites in addition to clinical symptoms. Meteorological and hydrological factors were measured daily. For each factors variation indices were estimated. Urbanization was yearly estimated assessing satellite imaging and field investigations. Principal component analysis was used for dimension reduction and factors combination. Lags between malaria incidences and the main components were assessed by cross-correlation functions. Generalized additive model was used to assess relative impact of different environmental components, taking into account lags, and modelling non-linear relationships. Change-point analysis was used to determine transmission periods within years.

**Results:**

Malaria incidences were dominated by annual periodicity and varied through time without modification of the dynamic, with no impact of the urbanization. The main meteorological factor associated with malaria was a combination of evaporation, humidity and rainfall, with a lag of 3 months. The relationship between combined temperature factors showed a linear impact until reaching high temperatures limiting malaria incidence, with a lag 3.25 months. Height and variation of the river were related to malaria incidence (respectively 6 week lag and no lag).

**Conclusions:**

The study emphasizes no decreasing trend of malaria incidence despite accurate access to care and control strategies in accordance to international recommendations. Furthermore, no decreasing trend was showed despite the urbanization of the area. Malaria transmission remain increase 3 months after the beginning of the dry season. Addition to evaporation versus humidity/rainfall, nonlinear relationship for temperature and river height and variations have to be taken into account when implementing malaria control programmes.

## Background

In Mali, malaria remains the leading cause of mortality and morbidity representing 42% of consultations in health centres [1]. The average national prevalence of malaria parasitaemia was 38% in 2010 and 52% in 2012–2013 in children under-5 with 10% in Bamako [2]. In urban and suburban areas where malaria is hypo-endemic with prevalence of less than 10% in children aged from 0 to 9, the burden of clinical malaria is classically low considering socio-economic, development and environmental factors [3]. The proportion of children under 5 years with fever in Bamako was 14.6% [4 **]** 2 years before and 8.2% in 2012 [2]. The use of insecticide-treated mosquito nets, the treatment of malaria cases by artemisinin-based combination therapy, and the chemoprevention of malaria in pregnant women are the main malaria control strategies in the area. The biological diagnosis of malaria is free at Sotuba Malaria Research Centre. Malaria cases treatment in children under 5 years and chemoprevention in pregnant women are also free. Insecticide-treated mosquito nets for children under 5 and pregnant women is distributed freely by the National Malaria Control Programme (PNLP). In general, Sotuba has not been affected by large movements of refugees during the study period from 2008 to 2012. Only the last year of the study coincided with the problem in the north of Mali, which did not significantly affect the study area. Urbanization has been reported as a factor which contributes to the decline in risk of malaria [5].

In Sahelian countries, such as Mali, rainfall is globally weak (200–1300 mm of rain) with variable period of drought. Meteorological factors are known to play an important role in the intra and inter-annual evolution of malaria vectors and incidences [6–9]. Among the numerous available meteorological factors only few of them are classically studied (cumulated rainfall, temperature, humidity). However, other specific factors, such as number of rainfall events, evaporation, temperature at different height, sunlight, wind speed, their variations and combinations are not assessed, nor the association with hydrologic factors, in the context of urbanization.

Malaria transmission is markedly heterogenic in endemo-epidemic areas according to the season and the year [10]. This variability has an impact on control activity planning against malaria. In the context of elimination of malaria, knowledge of malaria risk periods (high transmission periods) is essential for proper use of resources [11].

Observation data on malaria incidence and burden of clinical malaria are available in many health centres in Mali. The hypothesis was that the burden of clinical malaria cases may change according to environmental and urbanization risk factors. The aim of the study was to assess the impact of urbanization combined with meteorological and hydrological factors on the malaria dynamic in a growing suburban area along the Niger River.

## Methods

### Study site

**Fig. 1:**
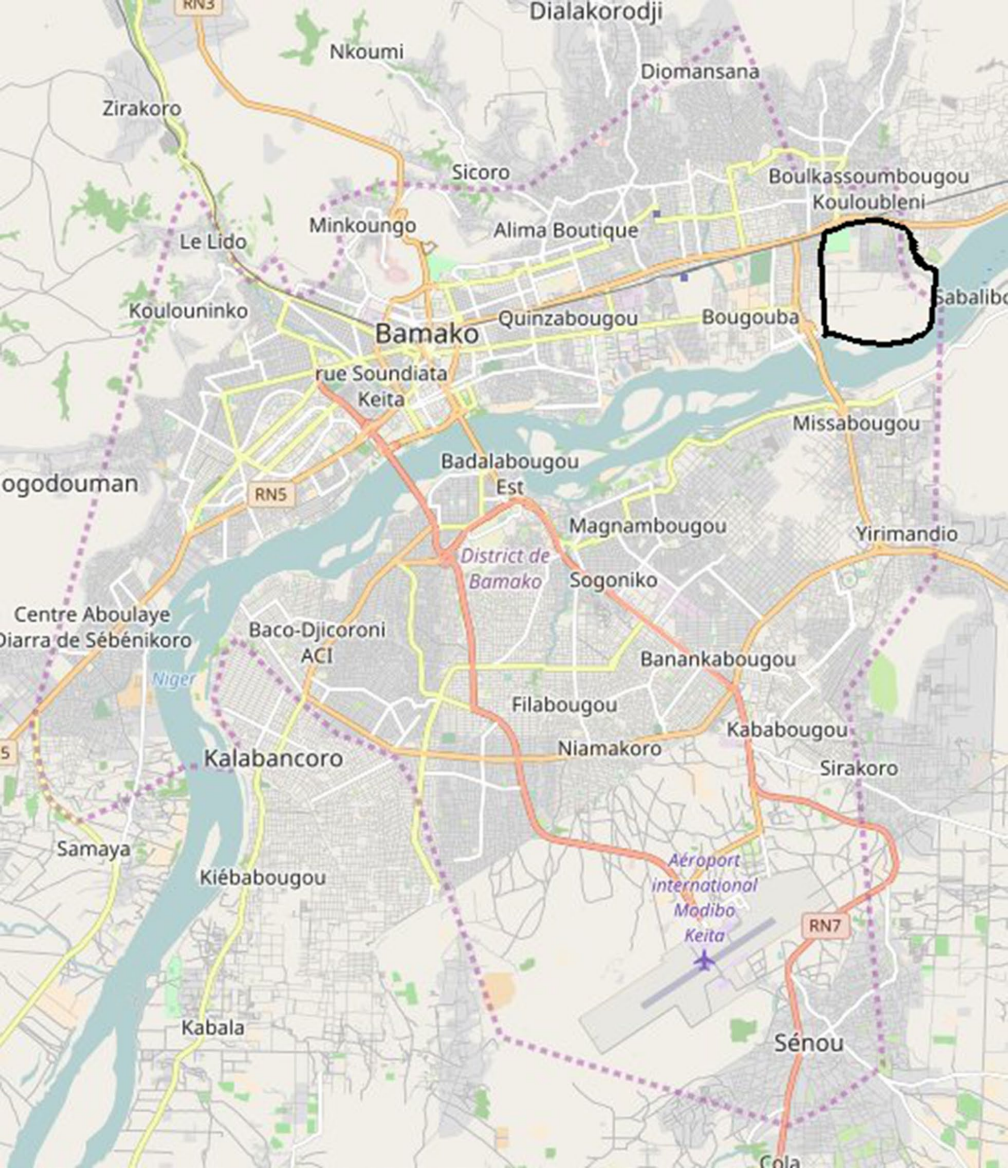
Map of Bamako located Sotuba

Sotuba is a suburban village located on the outskirts of Bamako on the bank of the Niger River (Fig. 1). Urbanization initially started in 1997, then implementation of consistent water supplies started in 2000, electricity in 2005, and roads and canals in 2008. In 2008, a census implemented in the area reported 6472 inhabitants (i.e. 607.7 inhabitants per square kilometre). Malaria transmission is mainly seasonal and occurs from May to October. The entomologic inoculation rate (EIR) has been estimated low (annual EIR < 15 infective bites per person) in 2000 [12]. Malaria incidence varied between 0.4 and 1.7 in children from 0 to 2 years in 2000 [12]. The Malaria Research and Training Centre (MRTC) team have established a very good relationship with the community, having completed many epidemiological studies and drug efficacy trials in the community since 1990, and a research medical clinic is maintained (Sotuba Malaria Research Centre, SMRC).

### Data collection

Population observational data were prospectively collected using the outpatient consultation and laboratory registers in the SMRC. From May 2008 to December 2012, all patients residing in Sotuba who consulted at the SMRC for clinical care with malaria compatible signs or symptoms were included in the study. SMRC was the only health centre in the village during the study period.

During outpatient consultations, clinical malaria was defined as the presence of any parasitaemia determined by microscopy and rapid diagnosis test (RDT) (without any changes in the diagnosis strategy during the study period) in addition to clinical variables as fever, headache, vomiting, diarrhoea, abdominal pain, nausea, body-aches, anorexia, convulsion, pallor, cough, myalgia and chills. Fever was defined as axillary temperature over 37.5 °C using an electronic thermometer.

In order to highlight the most important factors related to malaria incidence variations, the following environmental variables were measured daily in the village by the Rural Economic Institute (*Institut d’Economie Rurale*), located at Sotuba and the National Direction of Hydraulic: maximum inside temperature (t1M); minimum inside temperature (t1m); maximum ground temperature (t2M); minimum ground temperature (t2m); temperature at 10 cm (t10); temperature at 20 cm (t20); outside extreme humidity (hx); relative humidity (hr); evaporation using an experimental plug (evg); evaporation using an experimental pan (evn); cumulated rainfall (r); number of rainfall events (nre); sunlight (sl); tension value (tv); wind speed (ws); river height (rvh). The population density variation was estimated by counting buildings using satellite images from Google earth (© 2015 Google Inc. Menlo Park, California) from 2009 to 2012 based on the population census in 2008 (census done by MRTC team). Estimates were further completed by field investigations and interviews of village leaders to assess the number of inhabitants in the new buildings of the area.

### Statistical analysis

The analysis was divided into five main steps: (i) time series estimations, (ii) cofactors assessment and combination, (iii) lag assessment between cofactors and malaria incidence, (iv) multivariate analysis, and, (v) transmission period assessment.

I. The weekly cumulative malaria incidences, cumulated rainfall and rainfall events, and weekly mean, maximum, minimum, standard deviation (SD), range, and coefficient of variation (CV) of all the other meteorological and hydrological factors were estimated.
II. Meteorological and hydrological factors (19) and their variations were analysed through two principal component analysis (PCA). This method was used in order to combine factors, taking into account collinearity and for dimension reduction. The main components, determined by eigenvalues, were then characterized, for meteorological factors and hydrological factors.
III. Lags between each component and malaria incidence were assessed by cross-correlation function estimations, after modelling time processes for each time series, following the Box–Jenkins approach [13]. Each component showing a significant cross-correlation was then selected and shifted (according to the estimated lag) for the multivariate analysis.
IV. For the multivariate analysis, a regressive approach was developed, following a modelling strategy determined in order to take into account the different situations that may bias classical regression models:
  Taking into account over-dispersion in the number of malaria cases, a quasi-Poisson distribution was used, replacing a classical Poisson distribution for count data;
  In order to interpret the parameters (after exponential transformations) as incidence ratios, the population was used as an offset of the regression model after log transformation;
    Assessing non-linear relationships between malaria cases and each cofactors (the shifted components selected at the first step), spline functions were used;
  Taking into account collinearities between the different cofactors, we did use the main components of the previous PCAs (first step), which were, by definition, independent.
  A stepwise approach was also used based on the generalized Cross-validation criterion (GCV).
    These modelling strategy led us to the use of a general additive model (GAM), following Simon Wood recommendations [14].
V. The low and high transmission periods were determined by using change-point analysis based on mean and variance evolutions, and the PELT algorithm (Pruned Exact Linear Time) [15, 16].

All statistical analysis were performed by using the R 3.3.1 software (copyright © 2016 The R Foundation for Statistical Computing, Vienna, Austria) [14, 17].

## Results

### Population socio demographic characteristics

**Fig. 2:**
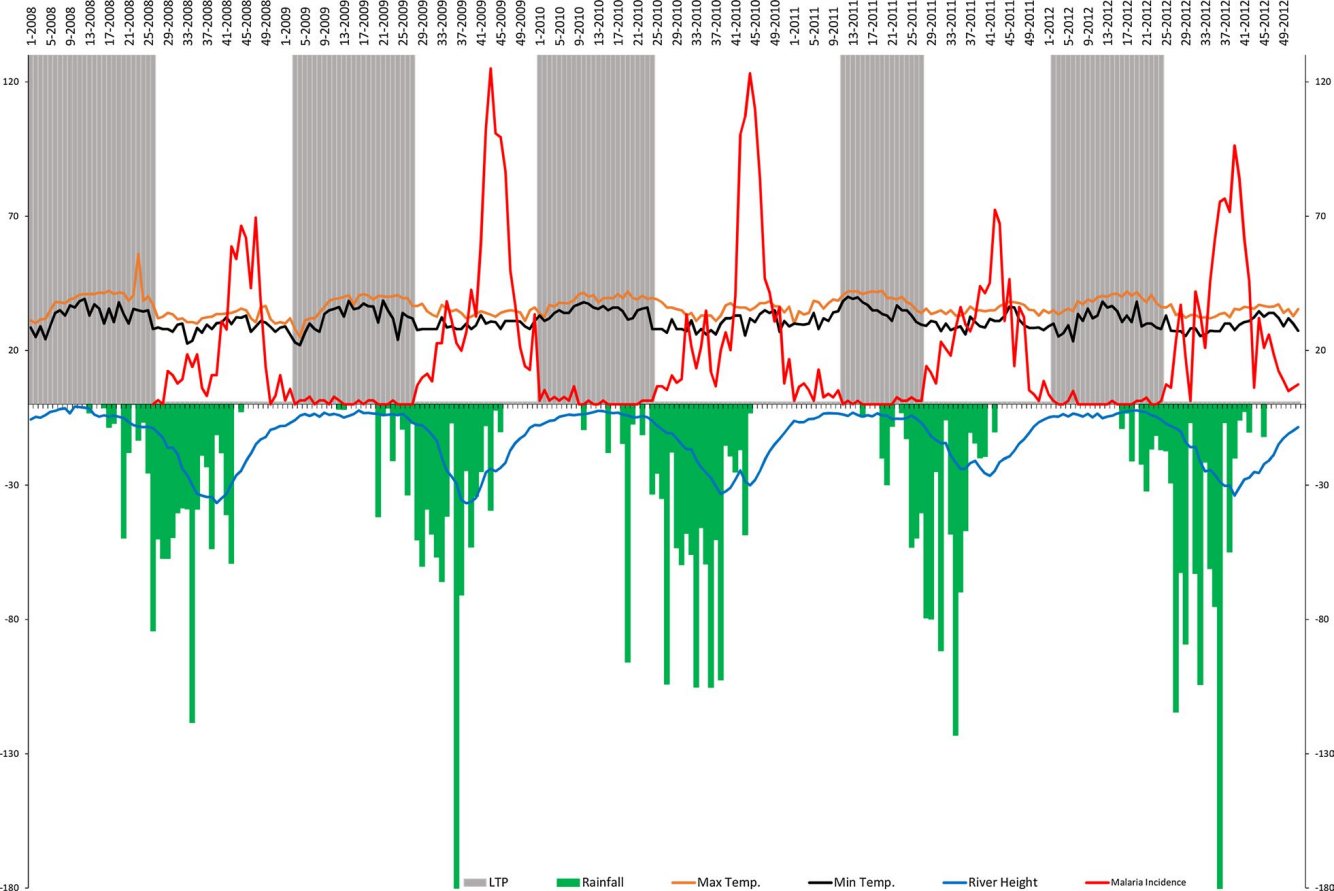
Malaria incidence time series, maximum temperature, minimum temperature, rainfalls and river height by week and by transmission period. The red line represents weekly malaria incidences, the orange and black lines represent respectively the weekly mean of maximum and minimum inside temperature (Mt1M; mt1m), the blue line represents the weekly mean of river height (rvh) and the green bar plot represents the weekly cumulative rainfall. The grey areas represent low transmission periods (LTP) according to the change point analysis

In total among 7075 consultations, 3286 malaria cases were notified by the SMRC from 2008 to 2012. The oscillations of malaria incidence time-series were dominated by the annual periodicity from 2008 to 2012. Malaria incidence varied intra and inter annually (Fig. 2). No modification of the dynamic of malaria incidence through time was observed, despite urbanization and a malaria control strategy in accordance with international recommendations. Population estimates increased linearly from 6472 to 8100, mainly in 2009 with a growth rate of 8.78%. The growth rate then decreased reaching 6.07% in 2010, 3.6% in 2011, and 4.71% in 2012.

The yearly rainfall varied from 1 year to another (Fig. 2). The highest yearly cumulative rainfall was seen in 2010 (1186.4 mm) and the lowest in 2011 (867 mm). The maximum (Mt1M) and minimum (mt1m) inside temperatures were similar throughout years, but varied intra-annually. The mean river height (rvh) changed annually (annual peak) and the dynamic remain similar from year to year. The years 2009 and 2010 showed the highest malaria incidence peaks, together with the highest population growth rates and the highest rainfall peaks.

### PCA of malaria risk factors

**Fig. 3:**
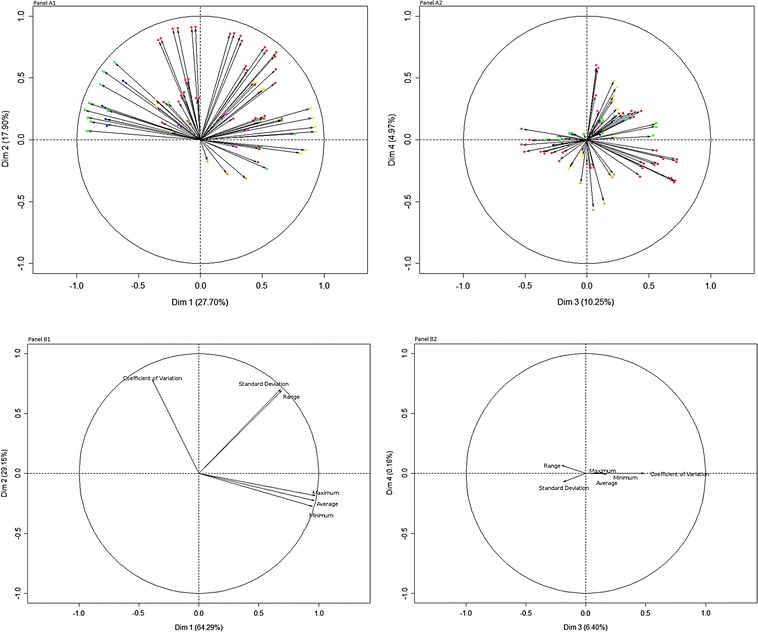
Principal component analysis of meteorological and hydrological factors. **A1** represents the two first components for meteorological factors, and **A2** the 3rd and 4th components. Red dots represent temperature factors, dark blue, rainfall, light blue, tension, green, humidity, yellow, evaporation, orange, sunlight, violet, wind speed. **B1** represents the two first components for hydrological factors, and **B2** the 3rd and 4th components

*Meteorological profiles* (Fig. 3A1, A2): the first four primary components explained 60.82% of the inertia. The first component (Met1) was constituted by evaporation versus humidity/rainfall. The second and third components (Met2 and Met3) represented mainly temperatures and temperature variations. The fourth components (Met4) was mainly constituted by sunlight and wind speed.

*River profiles* (Fig. 3 B1, B2): For river factors the first three primary components explained 99.84% of the inertia. The first component (Riv1) was constituted by the height of river and the second and the third components were constituted by the variations of river height (Riv2 and Riv3).

### Determination of PCA axes elapsing times by ARIMA of series according Box–Jenkins approach

**Fig. 4:**
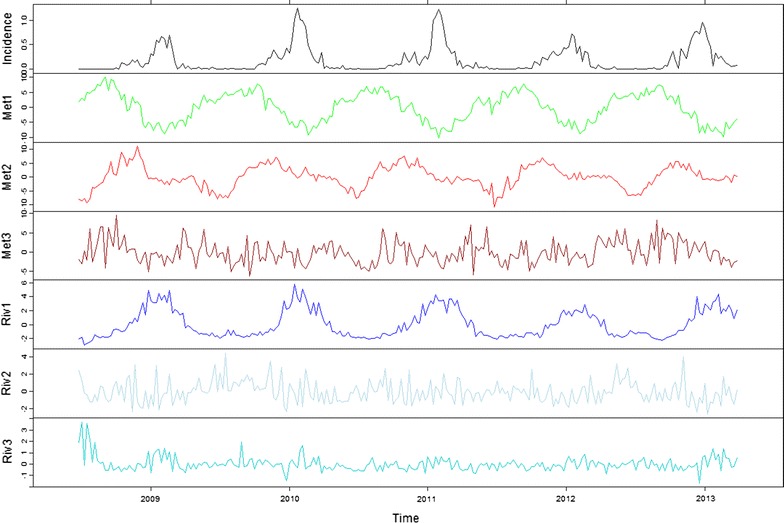
Incidence of malaria cases and main environmental components, shifted according to cross-correlation lags. The black line represents malaria incidence (%), the green line, evaporation/rainfall component (Met1, 12 weeks lag), the red line, temperature component (Met2, 13 weeks lag), the dark red line, temperature variation component (Met3, 5 weeks lag), the blue line, river height component (Riv1, 6 weeks lag), the light blue line, the first river height variation component (Riv2, no lag), the cyan line, the second river height variation component (Riv3, 7 weeks lag)

The cross-correlation functions showed that the main meteorological factors associated with malaria incidences was the combination of evaporation, humidity and rainfall (Met1 component), with a lag of 12 weeks, with a significant negative cross-correlation of − 0.14 (Fig. 4). The lag for temperatures component (Met2) was around 13 weeks, with a significant negative cross-correlation of − 0.13 (Fig. 4). For the temperature variation component (Met3), the lag was about 5 weeks (0.12), and for the sunlight/wind speed component (Met4) no significant cross-correlation was estimated (Fig. 4).

According to the hydrological factors, the river height component (Riv1) was significantly positively cross-correlated with malaria incidences (0.30) with a lag of 6 weeks (Fig. 4). The second component (first river variation component, Riv2) was significantly negatively cross-correlated with no lag (− 0.12), and the third component (second river variation component, Riv3) was significantly positively cross-correlated with a lag of 7 weeks (0.17) (Fig. 4).

### Modelling malaria incidences (GAM)

The environmental components significantly cross-correlated with malaria incidences (Met1, Met2, Met3, Riv1, Riv2, Riv3) were used, after shifted, as explaining factors of malaria incidences in the regressive GAM model, first in a univariate approach. The evaporation/rainfall (Met1) component showed a linear negative association (p < 0.0001) (Appendix: Table 1), and the river height component showed a linear positive association (p < 0.0001). The temperature component (Met2) showed a positive association (p = 0.0004) until reaching a threshold where a temperature increase did not increase malaria incidence. The temperature variation component (Met3) showed a non-significant negative relationship (p = 0.057) but not for lower values. The first river variation component (Riv2) showed significant slightly negative relationship, but only for the lowest values (p = 0.014). The second river variation component (Riv3), showed a nonlinear relationship, first negative, then positive, and then negative for higher values (p = 0.0003).

**Table 1:** General additive model results for univariate and multivariate analysis

	Univariate analysis				Multivariate analysis			
	EDF^a^	F^b^	p value	Dev (%)^c^	EDF^a^	F^b^	p value	Dev (%)^c^
Met1	2.291	51.89	< 0.0001	51.1	3.530	6.807	< 0.0001	68.3
Met2	3.632	5.464	0.0004	23.2	2.521	9.846	< 0.0001	
Riv1	3.645	44.34	< 0.0001	57.3	1.000	45.695	< 0.0001	
Riv2	2.346	3.938	0.0136	5.67	7.869	1.271	0.246	
Met3	2.287	2.505	0.0572	5.28				
Riv3	4.298	4.984	0.0003	11.8				

*Met1* evaporation/rainfall component, *Met2* temperature component, *Met3* temperature variation component, *Riv1* river height component, *Riv2* first river variation component, *Riv3* second river variation component

^a^EDF: estimated degrees of freedom for the model terms

^b^F: test statistics

^c^Dev: deviance explained

**Fig. 5:**
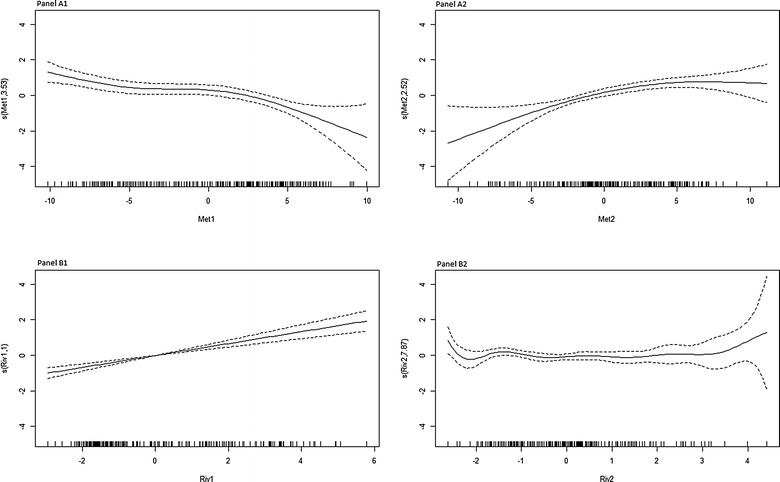
Association between malaria incidence and the meteorological and river components (GAM model). **A1** evaporation, humidity, rainfall component (Met1); **A2** temperatures component (Met2) (nonlinear relationship with malaria incidence); **B1** river height component (Riv1) and **B2** river height variation component (Riv2)

All components were analysed as factor of malaria incidence in the multivariate GAM model. After stepwise selection, only three components remained significant, and one non-significant component remained as a confounding factor. The evaporation/rainfall (Met1) component showed a linear negative association (p < 0.0001), and the river height component a linear positive association (p < 0.0001) (Fig. 5A1, B1). The temperature component (Met2) showed nonlinear relationship with malaria incidence, first positive, then null and slightly negative for higher values (Fig. 5A2). The river variation was non-significantly associated with malaria (p = 0.246), and the relationship wasn’t linear, first negative for the lowest values, then positive for the highest ones (Fig. 5B2).

### Change point analysis determining transmission periods

The change point analysis clearly showed the classical pattern of seasonal malaria, with low and high transmission periods (Fig. 1). Low transmission periods were not totally associated with dry seasons, and high transmission periods were not totally associated with rainy seasons, but less shifted with river height (Fig. 1).

## Discussion

Clinical malaria incidence and potential environmental risk factors, including temporal variations were investigated in suburban area of Bamako, Mali over 5 years from 2008 to 2012. Despite good access to care, diagnosis and treatment procedure in accordance to international recommendation, and the presence of a well-trained team, the temporal pattern of clinical malaria incidence did not change over years (Fig. 1). In particular, no reduction in the incidence of malaria was observed despite increasing urbanization compared to a phenomenon commonly reported in the literature that urbanization decrease the risk of malaria [5, 18]. The intra annual [12, 19–21] and inter annual [12, 20] variations of malaria are usual, with a pattern already described in many transmission settings. A lag between low transmission periods of malaria and dry seasons was observed, as well as between high transmission periods and rainy seasons. Conversely, a better concordance and a shorter lag were found between malaria incidence and river height patterns (6 weeks lag, Fig. 1).

The main meteorological factor associated with malaria is a combination of evaporation, humidity and rainfall (Fig. 5A1) described by several studies before. This linear correlation occurred with a lag of about 3 months. Even if in other area the lag between rainfall and malaria cases could be different, rainfall is not the only factor related to malaria transmission. Indeed, humidity, temperature, but also land cover/land use are important factors. As malaria transmission is known to be heterogeneous, according to geographical/environmental contexts, the lag difference between different areas is not surprising. Furthermore, Niakhar is a small city, and the data assessed by Ndiaye et al. aggregated informations from the whole rural area, showing millet and peanuts fields [22]. But Sotuba is a sub-urban area, very close to Bamako, the capital city. Note that, the SMRC team did not report any refugees camp near the Sotuba area during the study period.

The relationship between river high and rainfall at the same geographical area is not direct. Indeed, the Niger river high, in Bamako, is mainly due to rainfall in Conakry Guinea, at the source of the Niger river, and less related to the rainfall at Bamako. Furthermore, it is not the river high that is related to malaria incidence but also river variations, which could create, or destroy, breeding sites. This has also been observed in Bandiagara, Mali [10].

Most of the studies in the literature found that rainfall and humidity increase the risk of malaria by developing suitable breeding sites and increasing density of mosquitoes [9, 10, 20–28]. However, the positive association between humidity/rainfall and malaria incidence is inconsistent with other authors [29]. Furthermore, the length of the lag between rainfall and malaria incidence should be taken into account by prevention policies, including traveler recommendations on prophylaxis, malaria risk is still important, 3 months after rain stops.

The relationship between combined temperature factors was not linear (Fig. 5A2). This association was positively correlated and when temperature attempted a threshold where malaria incidence was constant, and then slightly decreased with temperature [29]. The lag time was 3.25 months between combined temperature factors and the incidence of malaria. This nonlinear association is concordant with malaria vector and parasite development cycles. This type of relationship is not taken into account in the major part of the literature. It can be explained by the existence of a temperature threshold fatal to the development of malaria vector (malaria transmission is optimal at 25 °C and decrease drastically at > 28 °C) [23, 30–32]. The main vector species are *Anopheles gambiae* s.l. and *Anopheles funestus*, with predominance of *An. gambiae*, which accounted for 99.7% of transmission [12]. Other studies found a positive linear association between malaria incidence and maximum temperature [9, 24, 27, 28, 33]. However, some studies observed negative association [29]. This apparent contradiction may be explained by the difference between the transmission periods, statistical methods (not exploring nonlinear relationships) and the diversity of the risk factors combined for the analysis.

Height of the Niger River was positively linearly correlated to malaria incidence (Fig. 5B1 and B2) with a lag of about 1.5 months. The variation of the river height plays an important role in the creation of anopheles breeding sites. When the river is full, the breeding sites on the banks of the river are completely submerged with water. However, the periods of flood recession are generally a source of breeding sites along the river. Humidity of the area could be driven also by river variation, and have an effect on mosquito survival [34]. Publications focused much more on the presence or absence of vectors or parasites carriers in the areas near the rivers [35, 36].

In conclusion, the present study emphasizes temporal variation of malaria incidence without change in the dynamics through the time. The lag between rainfall and malaria is classically acknowledged. These has to be considered for the implementation of malaria control strategies such as seasonal malaria chemoprevention, mass distribution of insecticide impregnated bed nets and mass sensitization campaigns to cover 3 months after rain stops. Nonlinear relationships for combined meteorological factors and other source of breeding sites (such as river) have to be taken into account when developing environmental based control strategies.

Furthermore, the persistence of clinical malaria during the low transmission period should be assessed, determining if different symptoms, clinical profiles and parasitaemia are associated with the different transmission periods.

## Appendix

See Table 1.

## References

[1] Ministère de la Santé. Annuaire Système Local d’Information Sanitaire (SLIS). Mali. 2012.

[2] Cellule de Planification et de Statistique (CPS/SSDSPF), Institut National de la Statistique (INSTAT/MPATP), INFO-STAT et ICF International (2014). Enquête Démographique et de Santé au Mali 2012–2013.

[3] Ceesay SJ, Bojang KA, Nwakanma D, Conway DJ, Koita OA, Doumbia SO (2012). Sahel, savana, riverine and urban malaria in West Africa: similar control policies with different outcomes. Acta Trop.

[4] Cellule de Planification et de Statistique du Ministère de la Santé (CPS/MS), Direction Nationale de la Statistique et de l’Informatique du Ministère de l’Economie, de l’Industrie et du Commerce (DNSI/MEIC) et Macro International Inc (2007). Enquête Démographique et de Santé du Mali 2006.

[5] Tatem AJ, Gething PW, Smith DL, Hay SI (2013). Urbanization and the global malaria recession. Malar J.

[6] Breman JG, Alilio MS, Mills A (2004). Conquering the intolerable burden of malaria: what’s new, what’s needed: a summary. Am J Trop Med Hyg.

[7] Al-Mansoob MA, Al-Mazzah MM (2005). The role of climate on malaria incidence rate in four governorates of Yemen. Med J Malays.

[8] Chaves LF, Koenraadt CJ (2010). Climate change and highland malaria: fresh air for a hot debate. Q Rev Biol.

[9] Reid HL, Haque U, Roy S, Islam N, Clements AC (2012). Characterizing the spatial and temporal variation of malaria incidence in Bangladesh, 2007. Malar J.

[10] Coulibaly D, Rebaudet S, Travassos M, Tolo Y, Laurens M, Kone AK (2013). Spatio-temporal analysis of malaria within a transmission season in Bandiagara, Mali. Malar J.

[11] Hay SI, Snow RW (2006). The malaria atlas project: developing global maps of malaria risk. PLoS Med.

[12] Dicko A, Sagara I, Diemert D, Sogoba M, Niambele MB, Dao A (2007). Year-to-year variation in the age-specific incidence of clinical malaria in two potential vaccine testing sites in Mali with different levels of malaria transmission intensity. Am J Trop Med Hyg.

[13] Shumway RH, Stoffer DS. Time series analysis and its applications. 3rd ed. Springer-Verlag: New York; 2011: XI, 596. p. 202.

[14] Wood SN (2006). Generalized additive models: an introduction with R.

[15] Killick R, Fearnhead P, Eckley IA (2012). Optimal detection of changepoints with a linear computational cost. JASA.

[16] Chen J, Gupta AK. Parametric statistical change point analysis. 2nd ed. Birkhauser Basel; 2012: XIII, 273.

[17] Lê S, Josse J, Husson F (2008). FactoMineR: an R package for multivariate analysis. J Stat Softw.

[18] Trape JF, Zoulani A (1987). Malaria and urbanization in Central Africa: the example of Brazzaville. Part III: relationships between urbanization and the intensity of malaria transmission. Trans R Soc Trop Med.

[19] Yé Y, Hoshen M, Kyobutungi C, Louis VR, Sauerborn R (2009). Local scale prediction of *Plasmodium falciparum* malaria transmission in an endemic region using temperature and rainfall. Glob Health Action.

[20] Stefani A, Hanf M, Nacher M, Girod R, Carme B (2011). Environmental, entomological, socioeconomic and behavioural risk factors for malaria attacks in Amerindian children of Camopi, French Guiana. Malar J.

[21] Donovan C, Siadat B, Frimpong J (2012). Seasonal and socio-economic variations in clinical and self-reported malaria in Accra, Ghana: evidence from facility data and a community survey. Ghana Med J.

[22] Ndiaye O, Le Hesran JY, Etard JF, Diallo A, Simondon F, Neil Ward M (2001). Variations climatiques et mortalité attribuée au paludisme dans la zone de Niakhar, Senegal, de 1984 à 1996. Cahiers d’études et de recherches francophones/Santé.

[23] Mordecai EA, Paaijmans KP, Johnson LR, Balzer C, Ben-Horin T, de Moor E (2013). Optimal temperature for malaria transmission is dramatically lower than previously predicted. Ecol Lett.

[24] Orlando PZ, Mikael A (2011). Spatial and temporal patterns of malaria incidence in Mozambique. Malar J.

[25] Gillioli G, Mariani L (2011). Sensitivity of *Anopheles gambiae* population dynamics to meteo-hydrological variability: a mechanistic approach. Malar J.

[26] Chaves LF, Satake A, Hashizume M, Minakawa N (2012). Indian Ocean Dipole and rainfall drive a Moran effect in East Africa malaria transmission. J Infect Dis.

[27] Zhang Y, Liu QY, Luan RS, Liu XB, Zhou GC, Jiang JY (2012). Spatial-temporal analysis of malaria and the effect of environmental factors on its incidence in Yongcheng, China, 2006–2010. BMC Public Health.

[28] Fang H, Shuisen Z, Shaosen Z, Hongju W, Linhua T (2011). Temporal correlation analysis between malaria and meteorological factors in Motuo County, Tibet. Malar J.

[29] Ali A, Monica CJ, Cesar K (2014). Modelling the effects of weather and climate on malaria distributions in West Africa. Malar J.

[30] Pascual M, Ahumada JA, Chaves LF, Rodo X, Bouma M (2006). Malaria resurgence in the East African highlands: temperature trends revisited. Proc Natl Acad Sci USA.

[31] Gosoniu L, Veta AM, Vounatsou P (2010). Bayesian geostatistical modeling of malaria indicator survey data in Angola. PLoS ONE.

[32] Zhou SS, Huang F, Wang JJ, Zhang SS, Su YP, Tang LH (2010). Geographical, meteorological and vectorial factors related to malaria re-emergence in Huang-Huai River of central China. Malar J.

[33] Yé Y, Louis VR, Simboro S, Sauerborn R (2007). Effect of meteorological factors on clinical malaria risk among children: an assessment using village-based meteorological stations and community-based parasitological survey. BMC Public Health.

[34] Yamana TK, Eltahir EAB (2013). Incorporating the effects of humidity in a mechanistic model of *Anopheles gambiae* mosquito population dynamics in the Sahel region of Africa. Parasite Vectors.

[35] Labbo R, Fandeur T, Jeanne I, Czeher C, Williams E, Arzika I (2016). Ecology of urban malaria vectors in Niamey, Republic of Niger. Malar J.

[36] Sissoko MS, van den Hoogen LL, Samake Y, Tapily A, Diarra AZ, Coulibaly M (2015). Spatial patterns of *Plasmodium falciparum* clinical incidence, asymptomatic parasite carriage and *Anopheles* density in two villages in Mali. Am J Trop Med Hyg.

